# Size-Dependent Nonlinear Optical Properties of Gd_2_O_2_S:Tb^3+^ Scintillators and Their Doped Gel Glasses

**DOI:** 10.3390/molecules27010085

**Published:** 2021-12-24

**Authors:** Long Chen, Cuiyu Wu, Zheng Xie, Chenghua Sun, Shuyun Zhou

**Affiliations:** 1 Key Laboratory of Photochemical Conversion and Optoelectronic Materials, Technical Institute of Physics and Chemistry, Chinese Academy of Sciences, Beijing 100190, China; chenlong174@mails.ucas.ac.cn (L.C.); wucuiyu19@mails.ucas.ac.cn (C.W.); zhengxie@mail.ipc.ac.cn (Z.X.); 2 University of Chinese Academy of Sciences, Beijing 100049, China

**Keywords:** scintillator, nonlinear optics, optical limiting, size-dependent

## Abstract

With the advancement of ultra-fast and high-energy pulsed laser output, lasers have caused serious harm to precision instruments and human eyes. Therefore, the development of optical limiting materials with a fast response, low optical limiting threshold, and high damage threshold are important. In this work, for the first time, it is reported that phosphors Gd_2_O_2_S:Tb^3+^(GOS) displays exceptional functionality in laser protection. GOS with sizes of 11 μm, 1 μm, and 0.45 μm are prepared. Based on the optical limiting and Z-scan technology systems under 532 nm and 1064 nm nanosecond laser excitation, the nonlinear optical properties of GOS are investigated. It is found that GOS exhibits outstanding optical limiting properties. In addition, the optical limiting response of GOS is size-dependent. Concerning the largest particle size, GOS has the best nonlinear optical response, while the precursor shows no nonlinear optical performance. Meanwhile, GOS doped gel glass also displays excellent optical limiting properties with high transmittance, which preliminarily validates the application of GOS and other scintillators in nonlinear optics and encourages more research to better realize the potential of GOS.

## 1. Introduction

Lasers have been rapidly developed and widely used in medical, material processing, and micromachining fields, stimulating a rising demand for laser protection [1,2,3]. Optical limiting (OL), in which the transmittance decreases with increasing laser intensity, is an ideal mechanism for the protection of eyes and sensitive optical devices from high-intensity lasers. In recent years, the OL properties of various materials have been investigated, among which two-dimensional nanomaterials [4], quantum dots [5], and organic polymers have displayed excellent OL performance [6,7]. Optical limiting materials with a low onset limiting and optical limiting threshold are anticipated to serve as candidates for constructing emerging photonic devices [8,9]. Therefore, it is necessary to discover new materials with low onset limiting thresholds, optical limiting thresholds, and large optical nonlinearities. Moreover, the methods by which size impacts the OL properties are worthy of studying, as the size evidently influences the bandgap and vacancies. For example, Liu et al. found that the larger TiS_2_ nanosheets exhibited better OL performance [10]. Zhou et al. demonstrated that the nonlinear optical (NLO) response of MoS_2_ and WS_2_ nanosheets were size-dependent [11]. Lu et al. reported that the size of WS_2_ nanosheets significantly influenced their NLO properties [12].

As a typical scintillator, Gd_2_O_2_S:Tb^3+^(GOS) phosphors have excellent fluorescence properties with a hexagonal crystal system. GOS has attracted wide attention in TV screens, cathode-ray tubes, and X-ray intensifying screens because of its excellent physical properties [13,14], such as high density and hard radiation stability [15,16]. There is much literature describing the electronic structure, physical and chemical properties, and toxicity of GOS [17,18,19]. However, the application of GOS for nonlinear optics has not been reported.

In this work, GOS of different sizes and high quality were synthesized via the precursor method and the high-temperature solid-phase method. The morphology and microstructure were characterized. In addition, the OL properties and size effect were explored. It was found that the GOS exhibited excellent optical limiting performance, and the larger size GOS possessed a more superior OL response. The mechanism included nonlinear absorption and nonlinear refraction. Finally, GOS was then introduced to the gel glass to form a solid sample and the OL performance was investigated. The wide range of sizes of GOS and the high transmittances of GOS/gel glass endow GOS with great potential for application in laser protection.

## 2. Results

### 2.1. Size-Dependent Characterization of Gd_2_O_2_S:Tb^3+^ Scintillators

**Figure 1:**
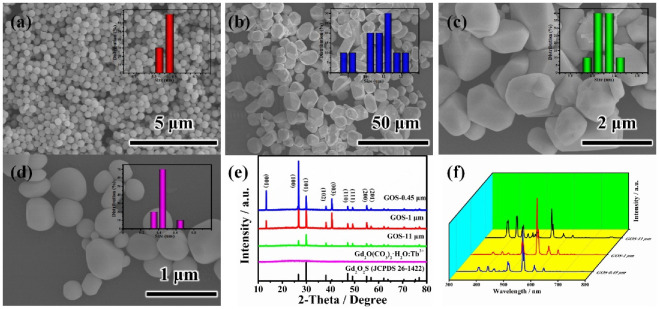
SEM images of (**a**) precursor Gd_2_O(CO_3_)_2_·H_2_O: Tb^3+^ (**b**–**d**) GOS. (**e**) XRD patterns of precursor Gd_2_O(CO_3_)_2_·H_2_O: Tb^3+^ and GOS. (**f**) photoluminescence (PL) spectra of GOS.

Figure 1b–d show the representative SEM images of the obtained Gd_2_O_2_S:Tb^3+^(GOS) with average diameters of 11 ± 1 μm (GOS-11 μm), 1 ± 0.1 μm (GOS-1 μm), and 0.45 ± 0.1 μm (GOS-0.45 μm). Figure 1e show the XRD patterns of the GOS phosphors, and all the diffraction peaks can be well-matched with the standard GOS pattern (JCPDS Card No. 26–1422). The obtained Gd_2_O(CO_3_)_2_·H_2_O: Tb^3+^ precursor indicates that spherical particles (in Figure 1a) show no apparent peaks (in Figure 1e), indicating an amorphous structure which was in good agreement with those reported in the literature [20].

Figure 1f show the photoluminescence (PL) spectra of the GOS phosphor. Under 280 nm excitation, sharp emissions ranging from 300 to 800 nm were observed for the GOS phosphor, which was due to transitions from ^5^D_3,4_ excited states to the ^7^F_J_ (J = 1–6) ground multiplets as assigned in the literature [1,21], with the ~544 nm green emission (^5^D_4_ → ^7^F_5_ transition) being the strongest. Among the three samples, GOS-1 μm had the strongest luminescence intensity, GOS-0.45 μm was the next, and GOS-11 μm was the weakest.

### 2.2. Size-Dependent Optical Limiting Properties of Gd_2_O_2_S:Tb^3+^ Scintillators

**Figure 2:**
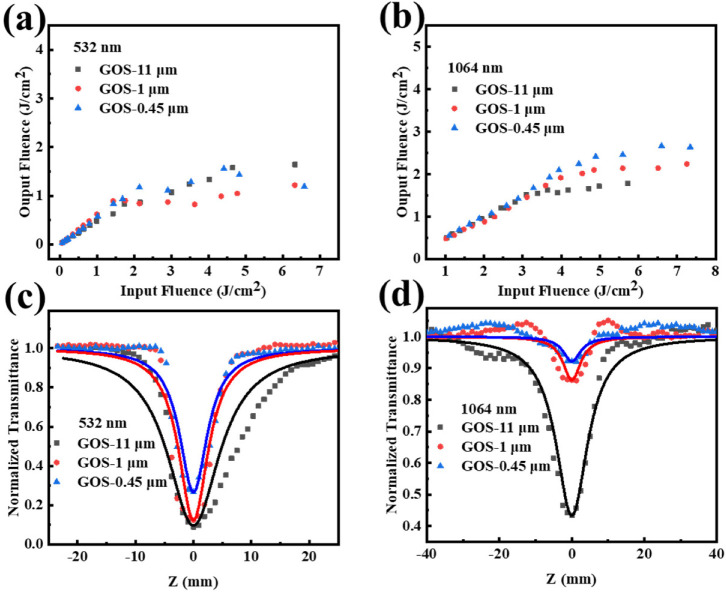
Optical limiting curve of GOS with different sizes in the solution state at (**a**) 532 nm; (**b**) 1064 nm; Open-aperture Z-scan data and theoretically fitted curves (solid curves) of GOS-(11 μm–0.45 μm) at (**c**) 532 nm (**d**) 1064 nm.

The optical limiting properties of the as-prepared samples were investigated at 532 nm and 1064 nm excitation with a laser pulse of 10 ns. The different sizes of GOS were dispersed in glycerinum, and the transmittance was tuned to 70% at the corresponding wavelength. It can be seen from Figure 2 that GOS with different sizes exhibit optical limiting performance at 532 nm and 1064 nm. That is, all GOS exhibit an obvious optical limiting response, which means the GOS can allow for low-intensity light but effectively suppress high-intensity light. Similarly, the precursor Gd_2_O(CO_3_)_2_·H_2_O: Tb^3+^ shows no signal at the same condition (Figure S1), indicating that GOS has certain potential applications in a wide spectrum. The onset limiting threshold (F_ON_, defined as the input energy density at which the output energy density starts to decrease) and limiting threshold (F_OL_, defined as the input fluence where the transmittance reduces to half of the linear transmittance) are two vital parameters to evaluate the optical limiting performance. Materials with low F_ON_ and F_OL_ are predicted to be promising candidates in optical limiting applications. The variations of normalized transmittance with incident intensity are shown in Figure 2. For 532 nm excitation, the F_ON_ of GOS-11 μm, GOS-1 μm, and GOS-0.45 μm were 0.99, 1.43, and 1.69 J·cm^−2^, and the F_OL_ were 2.17, 2.90, 3.53 J·cm^−2^, respectively. For 1064 nm excitation, the F_ON_ of GOS-11 μm, GOS-1 μm, and GOS-0.45 μm were 2.8, 3.98, and 4.46 J·cm^−2^, and the F_OL_ were 4.18, 5.59, and 5.59 J·cm^−2^, respectively. The GOS-11 μm has the lowest F_ON_ and F_OL_ at two wavelengths (Table S1), revealing that the larger the GOS display, the better the OL performance. Moreover, Table S2 presents the comparison of the OL properties of three GOS samples and some typical materials, including Sb nanosheets, graphene, and graphene-ormosil [10,22,23,24,25]. It can be seen that the three GOS samples in glycerol have certain OL properties.

The nonlinear absorption of GOS was studied by Z-scan. As shown in Figure 2c, the normalized transmittance of the three samples decreased as the incident intensity increased (Z → 0); GOS exhibited decreased transmittance near the zero Z-position as the lateral size increased from 0.45 μm to 11 μm. It is worth noting that glycerinum has no nonlinear absorption, and the NLO effect originates from the contribution of the GOS (Figure S2).

**Figure 3:**
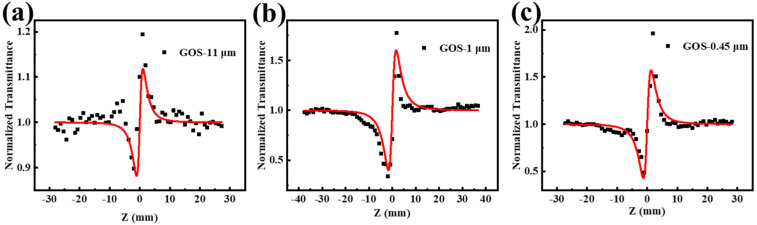
Closed aperture Z-scan data and theoretically fitted curves at 532 nm (solid curves) (**a**) GOS-11 μm, (**b**) GOS-1 μm, (**c**) GOS-0.45 μm.

The nonlinear refraction was investigated by the closed-aperture Z-scan approach. Figure 3 show that all the GOS samples exhibit self-focusing signals at 532 nm, suggesting positive refractive indices. The incident fluence of GOS-11 μm, GOS-1 μm, and GOS-0.45 μm are 0.9, 2.6, and 0.9 J·cm^−2^. The nonlinear refraction signals show that three samples have a self-focusing effect. These results prove that the GOS material has nonlinear optical properties, which are caused by the GOS material itself and the comprehensive results of a variety of effects, such as nonlinear absorption and nonlinear refraction.

### 2.3. The Gd_2_O_2_S:Tb^3+^ Scintillators Doped Gel Glass

**Figure 4:**
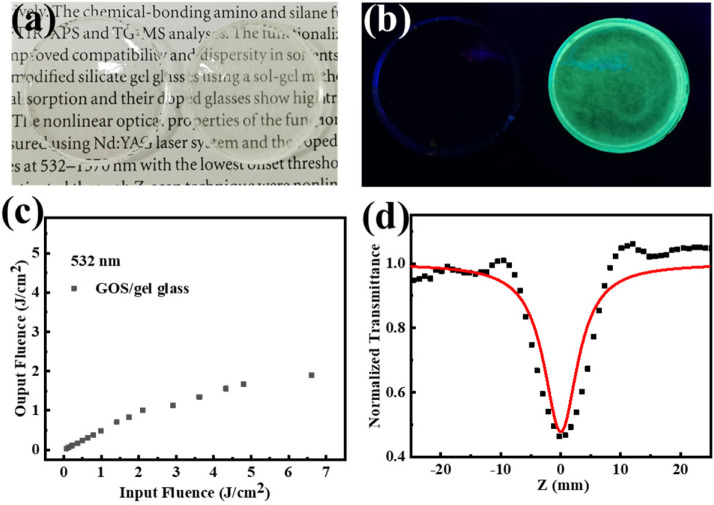
(**a**) Photographs of blank glass (left) and GOS/gel glass (right); (**b**) Photographs of blank glass (left) and GOS/gel glass under UV lamp; (**c**) Optical limiting curve of GOS/gel glass at 532 nm; (**d**) Open-aperture Z scan of GOS/gel glass at 532 nm.

To illustrate the practicality of GOS in optical limiting, GOS doped gel glass was prepared using a simple sol-gel method. The photographs of blank gel glass and GOS/gel glass with the dope concentration of 0.5 wt% are shown in Figure 4a.

The transmittance spectra in Figure S4 show that the GOS/gel glass possesses high transmittances in the visible and near-infrared region. The high transmittance of >60% at 350–1300 nm indicates practical applications. As shown in Figure 4b, the GOS/gel glass shows bright green fluorescence while the blank gel glass is transparent under the illumination of the UV lamp, revealing the uniform dispersion of GOS in the gel glass. The optical limiting performance of GOS/gel glass at 532 nm was investigated, and the results show the GOS/gel glass exhibits good optical limiting properties. Figure 4d show the result of the open aperture Z-scan of the GOS/gel glass at 532 nm, while the blank gel glass shows no signal (Figure S3). The F_ON_ and F_OL_ of GOS/gel glass were 1.74 and 3.61 J·cm^−2,^ respectively, indicating that the glass can serve as a solid-state optical limiter. 

## 3. Materials and Methods

The following chemicals were used in this work: GdCl_3_·6H_2_O (99.9%), TbCl_3_·6H_2_O (99.99%) urea (99.5%), S (99.99%), Tb_4_O_7_ (99.999%), and Gd_2_O_3_ (99.9%); all were purchased from Aladdin. Na_2_CO_3_ of analytical purity were obtained from Sinopharm Chemical Reagent Co., Ltd. All reagents were analytically graded and used without further purification. Deionized water was used as a solvent throughout the experiment.

### 3.1. Synthesis of Precursor Gd_2_O(CO_3_)_2_·H_2_O: Tb^3+^ Particles

First, GdCl_3_·6H_2_O, TbCl_3_·6H_2_O, and urea were added to deionized water with constant stirring. Next, the transparent solutions were maintained at 91 °C for a period of 4 h, leading to the formation of white precipitates. Subsequently, the white precipitates were collected by suction filtration and washed three times with deionized water. This was followed by drying in a vacuum oven at 80 °C overnight. Finally, precursor Gd_2_O(CO_3_)_2_·H_2_O: Tb^3+^ powders were obtained.

### 3.2. Synthesis of Gd_2_O_2_S: Tb^3+^ Particles

Precursor Method: The precursor Gd_2_O(CO_3_)_2_·H_2_O: Tb^3+^, S, and Na_2_CO_3_ were mixed thoroughly. Then, the the mixed raw materials were placed in a crucible, which was calcined at 900 °C with 5 °C·min^−1^ rate for 4 h in a reducing atmosphere (in 8 vol%H_2_/Ar). The product obtained was washed with deionized water and dilute hydrochloric acid. The phosphor powders were finally obtained after drying at 80 °C overnight.

High-temperature solid-phase method: The Gd_2_O_3_, Tb_4_O_7_, S, and Na_2_CO_3_ were mixed thoroughly. The following experimental steps are the same as Precursor Method.

### 3.3. Preparation of GOS Gel Glass:

GOS gel glass was prepared by the sol-gel method, which was via the hydrolysis and polycondensation of MTES in acidic conditions (acetic acid, pH = 2.5). The molar ratio of MTES:water:ethanol was 1:3.5:12.05. The mixture was stirred overnight and the solvents were removed by rotary steaming. Then the GOS was added to the sol and was stirred evenly. Lastly, the GOS doped gel was cast into polypropylene cells and dried to obtain the GOS gel glass.

### 3.4. Characterization

X-ray diffraction (XRD) was performed on a D8 Advance X-ray diffractometer (Bruker, Germany) with Cu Kα radiation (λ = 1.54056 Å) at 40 kV and 40 mA. Scanning electron microscopy (SEM) images were obtained on a Hitachi S-4800 Scanning Electron Field Emission Microscope. The emission spectra of the products were recorded by a Carry Eclipse (Agilent Technologies, California, CA, USA). All measurements were performed at room temperature (RT). The NLO properties at 532 and 1064 nm were investigated using a Z-scan setup (Nd: YAG laser, pulse duration: 10 ns, repetition rate: 10 Hz). The beam waist radius of 532 and 1064 nm are 13.5 and 27 μm for the Z scan setup, respectively. The optical limiting performance of GOS with different sizes in the solution state and the gel glass state were performed at 532 nm and 1064 nm excitation with the waist radius of 25 μm and 27 μm.

## 4. Conclusions

In summary, we have successfully prepared different lateral sizes of GOS (11 μm, 1 μm, and 0.45 μm) via the precursor method and the high-temperature solid-phase method. The study reported the first nonlinear optical properties of GOS and their size effects. GOS exhibited excellent optical limiting performance, and the larger GOS performed better. Subsequently, the GOS was doped into gel glass. The obtained solid sample possessed high transmittance at a dope concentration of 0.5 wt% and showed an outstanding optical limiting response, revealing their potential in developing nonlinear and optoelectronic materials and devices.

## Data Availability

The data presented in this study are available in Supplementary Materials.

## Supplementary Materials

The following supporting information can be downloaded, Figure S1: Z-scan data of precursor Gd_2_O(CO_3_)_2_·H_2_O: Tb^3+^, Figure S2: Z-scan data of glycerol, Figure S3: Z-scan data of blank gel glass, Figure S4: Transmission spectra of GOS /gel glass, Table S1. The onset limiting threshold and limiting threshold of GOS, Table S2. Comparison of optical limiting properties of different materials and particle sizes.

## Sample Availability

Samples of Gd_2_O_2_S:Tb^3+^ scintillators are available from the authors.
