# Supplementary material for: Size-Dependent Nonlinear Optical Properties of Gd2O2S:Tb3+ Scintillators and Their Doped Gel Glasses
Source: Molecules. 2021 Dec 24;27(1):85. doi: 10.3390/molecules27010085 (PMC8746479; doi:10.3390/molecules27010085)
Supplement: Supplementary file 1 [file molecules-27-00085-s001.zip › molecules-1465679-supplementary.pdf]

Size-dependent nonlinear optical properties of $Gd_2O_2S:Tb^{3+}$ scintillators and their doped gel glasses

Long Chen[1,2,†], Cuiyu Wu[1,2,†], Zheng Xie[1], Chenghua Sun[1,*], Shuyun Zhou[1,*]

[1] Key Laboratory of Photochemical Conversion and Optoelectronic Materials, Technical Institute of Physics and Chemistry, Chinese Academy of Sciences, Beijing, 100190, China.

[2] University of Chinese Academy of Sciences, Beijing 100049, China

* Corresponding author: sunchenghua@mail.ipc.ac.cn, zhou_shuyun@mail.ipc.ac.cn

† These authors contributed equally to this work.

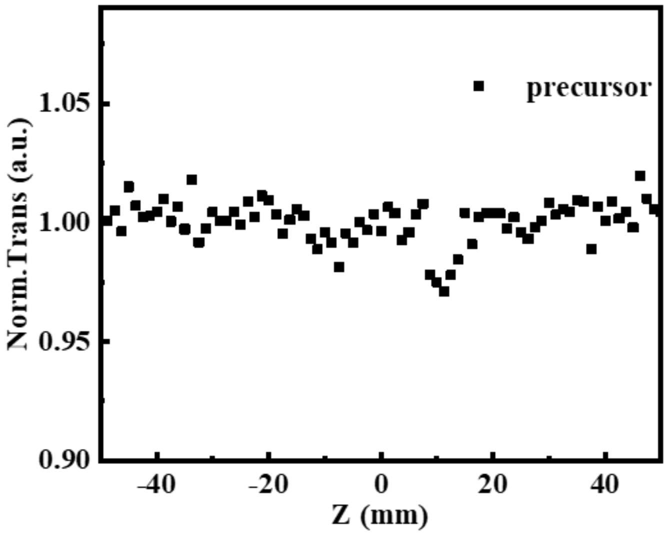

Figure S1. Z-scan data of precursor $Gd_2O(CO_3)_2 \cdot H_2O : Tb^{3+}$.

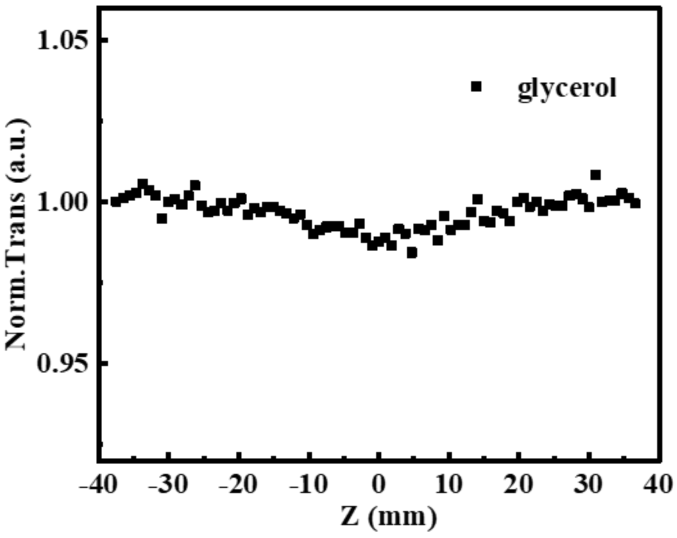

Figure S2. Z-scan data of glycerol.

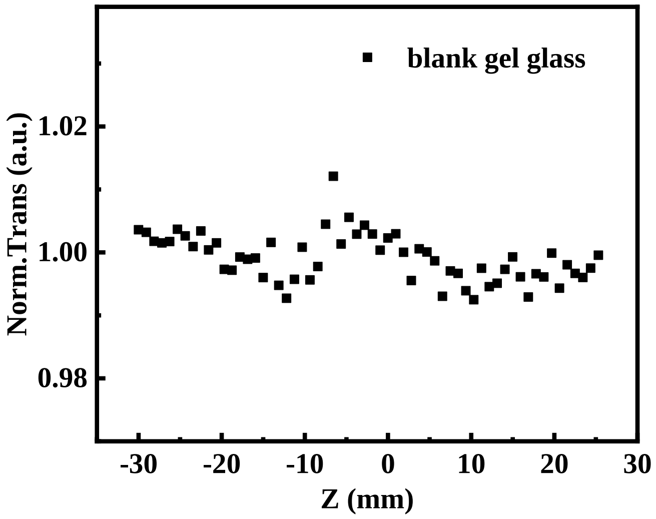

Figure S3. Z-scan data of blank gel glass.

Table S1. The onset limiting threshold and limiting threshold of GOS.

samples	λ (nm)	F_{ON} (J·cm^{-2})	F_{OL} (J·cm^{-2})
GOS-11 μm	532	0.99	2.17
GOS-1 μm		1.43	2.90
GOS-0.45 μm		1.69	3.53
GOS-11 μm	1064	2.8	4.18
GOS-1 μm		3.98	5.59
GOS-0.45 μm		4.46	5.59

Table S2. Comparison of optical limiting properties of different materials and particle sizes.

Material	size	Solvent/Matrices	laser parameters	F_{ON}（J/cm^2）	F_{OL}（J/cm^2）	literature
$WS_4Cu_4Cl_2(py)_6$	/	DMF	532 nm 8 ns	/	0.10	[22]
TiS_2 nanosheet	350 nm	PMMA glasses	532 nm 7 ns	0.067	0.8	[10]
Sb nanosheet	Several hundred naometers	isopropyl alcoho	532 nm 1.8 ns	0.162	1.37	[23]
Graphene	A few micrometers	NMP	532 nm 6 ns	0.44	15.15	[24]
Graphene-Ormosil	/	gel glasses	532 nm 6 ns	0.03	/	[25]
GOS	0.45 μm	glycerol	532 nm 10 ns	1.69	3.53	This work
	1 μm			1.43	2.90	
	11 μm			0.99	2.17	

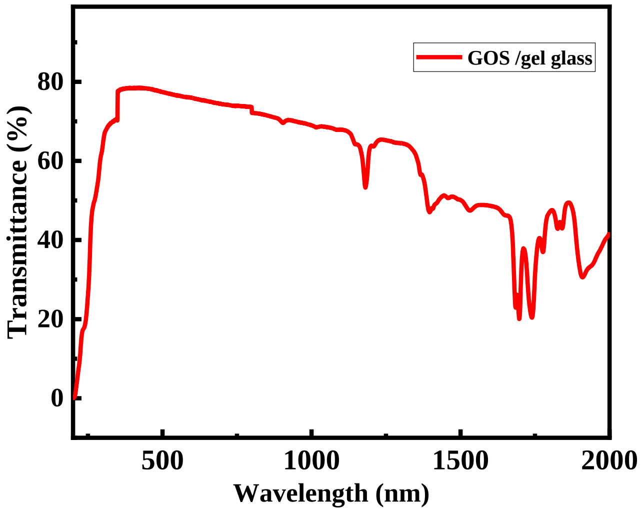

Figure S4. Transmission spectra of GOS /gel glass.
